# Consistency of Judging Under the World Boxing 10-Point Must Scoring System: Single-Judge and Five-Judge Aggregate Reliability Across the 60 kg, 75 kg, and 90+ kg Categories at the 2025 World Boxing Championships

**DOI:** 10.3390/sports14090403

**Published:** 2026-09-14

**Authors:** Gašper Munda, Goran Kuvačić, Sara Besal, Damir Karpljuk, Marco Batista, Łukasz Rydzik, Jožef Šimenko

**Affiliations:** 1 Faculty of Sport, University of Ljubljana, 1000 Ljubljana, Slovenia; gasper.munda1@gmail.com (G.M.); sara.besal@fsp.uni-lj.si (S.B.); damir.karpljuk@fsp.uni-lj.si (D.K.); 2 Faculty of Kinesiology, University of Split, 21000 Split, Croatia; goran.kuvacic@kifst.eu; 3 Sport Physical Activity and Health Research & Innovation Center (SPRINT), Polytechnic University of Castelo Branco (UPCB), 6000-084 Castelo Branco, Portugal; marco.batista@ipcb.pt; 4 Department of Sport Theory and Motor Skills, Institute of Sports Sciences, University of Physical Culture in Kraków, 31-571 Krakow, Poland; lukasz.rydzik@awf.krakow.pl

**Keywords:** world boxing, officiating, inter-judge reliability, combat sports, generalizability theory, scoring reliability

## Abstract

**Background**: Reliable judging is essential to the fairness of elite boxing, yet inter-judge reliability under the current World Boxing 10-Point Must Scoring System remains largely unexplored. This study aimed to evaluate inter-judge reliability across rounds and weight categories at the 2025 World Boxing Championships. **Methods**: A retrospective observational analysis included 94 men’s bouts in the 60 kg (*n* = 42), 75 kg (*n* = 23), and 90+ kg (*n* = 29) categories. Judge-level round scores were analysed using linear mixed-effects variance-component models, with bout-round target and judge identity specified as random effects. Reliability was estimated for individual judge ratings (R_single_) and an aggregate measurement based on five judge ratings (R_panel(5)_). Pairwise differences across rounds and weight categories were evaluated using bout-level bootstrap procedures with Holm adjustment for multiple testing. **Results**: Across all bouts, R_single_ was 0.617 (95% CI: 0.521–0.693) in Round 1, 0.634 (95% CI: 0.539–0.709) in Round 2, and 0.687 (95% CI: 0.597–0.754) in Round 3. The corresponding R_panel(5)_ estimates were consistently higher at 0.889 (95% CI: 0.845–0.919), 0.897 (95% CI: 0.854–0.924), and 0.916 (95% CI: 0.881–0.939), respectively. No statistically significant differences in reliability were detected across rounds or between weight categories after Holm adjustment. Overall, 98.2% of judge-round scores were 10:9 and 68.9% of bouts decided by judges’ scores ended in unanimous 5:0 decisions. **Conclusions**: Reliability was consistently higher when five judge ratings were aggregated than when individual judge ratings were considered. No statistically significant evidence of systematic differences in reliability across rounds or between the examined weight categories was detected.

## 1. Introduction

Judging, unfortunately, has long been one of the most debated aspects of elite international boxing. Throughout the history of sport, controversial decisions have repeatedly generated criticism from athletes, coaches, officials, and spectators, raising concerns about the fairness and credibility of competition [1,2]. These concerns have been reinforced by the independent McLaren Report, which identified serious integrity issues within Olympic boxing officiating [3]. In response, substantial reforms have been introduced, including the establishment and subsequent IOC recognition of World Boxing as the international federation governing boxing within the Olympic Movement, as well as revised governance and officiating procedures to enhance transparency, integrity, and consistency in judging [4]. Although recent reforms have introduced measures to strengthen the selection, certification, evaluation, and independent oversight of referees and judges [5,6], empirical evidence on inter-judge reliability under the current 10-Point Must Scoring System remains limited in elite international boxing.

Elite international boxing presents a particularly demanding environment for officiating. Under the current World Boxing Competition Rules, every bout is evaluated independently by five judges using the 10-point must-scoring system [7], which was introduced in 2013 [8]. Under the current rules, every round must have a declared winner and no round can be scored as a draw, while each round is scored using three levels: 10–9 for a close round, 10–8 when there is a clear winner, and 10–7 in cases of total dominance, with seven points representing the minimum score that can be awarded to the losing boxer. At the end of the bout, each judge determines the winner based on the total scores across all rounds. A unanimous decision is reached when all five judges favour the same boxer (5:0), while a split decision occurs when three or four judges favour one boxer (4:1; 3:2). If a judge’s total scores are tied at the end of the bout, a tie-break is required only under specific conditions defined by the World Boxing Competition Rules, including situations in which the remaining judges do not provide a decisive outcome. In exceptional cases, a judge may retain a tied total scorecard when the remaining judges already provide a decisive outcome, resulting in a 4:0 decision [7]. Round scores are awarded according to the number and quality of scoring blows landed on the target area, technical and tactical superiority, and competitiveness. Judges must continuously evaluate numerous offensive and defensive actions in real time. Elite boxers initiate offensive or defensive actions approximately every 1.4 s, performing around 20 punches, 2.5 defensive movements, and 47 vertical hip movements per minute [9], placing considerable perceptual and cognitive demands on officials. However, these activity patterns are not uniform across weight categories [10]. Previous research has demonstrated weight-category-specific differences in the temporal and technical–tactical structure of Olympic boxing bouts. In an analysis of flyweight, middleweight, and super heavyweight bouts at the Tokyo 2020 Olympic Games, researchers reported significant differences in the total number of punches and the areas of the ring where punches were thrown, with flyweight boxers exhibiting the highest number of actions [9]. Such differences may also be relevant to judging, as the activity patterns associated with different weight categories can shape what judges see and need to process during a round. Judges do not base their decisions solely on the number of punches thrown; they consider several aspects of performance. Previous research showed that punch accuracy and fighting style correctly classified 85% of bout outcomes, highlighting the multidimensional nature of judging in boxing [11]. As the technical and tactical characteristics of a bout vary across weight categories, the superiority of one boxer may also be more or less apparent to individual judges. Examining inter-judge agreement across the lightweight, middleweight, and heavyweight categories may therefore help establish whether judging consistency changes across different competitive contexts.

The combination of these multidimensional scoring demands and weight-category-specific activity patterns may therefore influence how consistently different judges evaluate the same bout. Examining judging reliability across lightweight, middleweight, and heavyweight contests may provide a more complete picture of how consistently the current judging system performs across different competitive contexts.

Assessing inter-judge consistency is an established approach to evaluating officiating systems in sports where performance is determined, at least in part, through subjective evaluation, and is particularly relevant following changes to scoring rules or judging procedures [12,13,14]. Inter-judge agreement provides information on how consistently officials interpret and apply the same scoring criteria and has consequently been examined across a range of judged sports, including combat sports, gymnastics, and other summer and winter Olympic disciplines [12,13,14,15,16,17,18]. Such analyses have shown that judging consistency may depend not only on the judges themselves, but also on how clearly scoring criteria are defined and applied. In combat sports, for example, Myers et al. [12] found greater agreement among judges when scoring criteria were more clearly defined and operationalised, suggesting that the structure and interpretation of the scoring framework can directly affect judging consistency.

Despite the extensive use of the 10-Point Must System in Olympic boxing, evidence on inter-judge reliability under the current World Boxing framework remains limited. Recent research has renewed interest in boxing officiating by examining judging bias, the scoring system’s limitations, and alternative approaches to aggregating judges’ scores, and by proposing modifications to improve the fairness and transparency of the judging process [1,2]. However, these studies address different aspects of judging and do not directly establish the extent to which judges agree when independently scoring the same rounds. Consequently, the consistency with which judges apply the current World Boxing scoring criteria during elite international competition remains unclear. To our knowledge, no previous study has evaluated inter-judge reliability using official round-level scores from an elite World Boxing competition. Establishing the degree of agreement between judges may therefore provide an important benchmark for evaluating the consistency of the current judging system and informing future developments in officiating practice, judge education, and governance.

Accordingly, the aims of this study were (1) to evaluate inter-judge reliability under the World Boxing 10-Point Must Scoring System and determine whether judging reliability differed across rounds using official scores from the 2025 World Boxing Championships, and (2) to examine whether inter-judge reliability varied across rounds within the purposively selected lightweight (60 kg), middleweight (75 kg), and heavyweight (90+ kg) categories, and whether reliability differed between these weight categories.

## 2. Materials and Methods

### 2.1. Study Design and Sample

This study employed a retrospective observational design to examine inter-judge reliability in elite international boxing under the World Boxing 10-Point Must System from the 2025 World Boxing Championships held in Liverpool, United Kingdom (www.worldboxingliverpool.com).

The study included all men’s bouts contested from three purposively selected weight categories: 60 kg, 75 kg, and 90+ kg. These categories were selected to enable a spectrum-based comparison of inter-judge agreement. The selection was informed by previous performance analyses in boxing, taekwondo, and judo, which compared categories distributed across the weight range and identified weight-category-specific differences in temporal, activity, and technical–tactical profiles [9,19,20]. Because such differences may alter the perceptual and cognitive demands placed on judges, examining categories from distinct portions of the weight spectrum provided a basis for assessing whether inter-judge agreement varied according to weight category. A total of 94 bouts were included, comprising 42 bouts in the 60 kg category, 23 bouts in the 75 kg category, and 29 bouts in the 90+ kg category. The analysed bouts involved 97 boxers: 43 in the 60 kg category, 24 in the 75 kg category, and 30 in the 90+ kg category. As boxers progressing through the tournament could participate in multiple bouts, the number of bouts does not correspond directly to the number of individual boxers.

The number of bouts included in individual analyses varied according to bout outcome and the number of rounds completed. Analyses of final judges’ decisions were restricted to bouts decided by judges’ scores, whereas round-specific inter-judge reliability analyses included only rounds for which complete scores from all five judges were available. Three bouts were terminated during the first round before a complete set of judges’ scores was available and therefore did not contribute to the round-specific reliability analyses. Consequently, 91 bouts provided complete scoring data for Rounds 1 and 2, while 86 bouts provided complete scoring data for Round 3. For the reliability analyses, each bout-round was treated as a single analytical target. Judges’ scores were coded as signed score margins relative to a fixed corner, thereby preserving both the direction and magnitude of each judge’s round-level decision while avoiding duplicate representation of the two structurally complementary boxer scores. All athletes competed in the men’s Elite division under World Boxing regulations. The study was based exclusively on the secondary analysis of publicly available official competition records. No participants were recruited or contacted, no intervention was carried out, and no private or confidential information was accessed. On this basis, the study did not involve prospective human-participant research, and ethical approval and informed consent were not required [21,22,23].

### 2.2. Data Sources and Data Collection

Data were obtained from the official website of the 2025 World Boxing Championships in Liverpool (www.worldboxingliverpool.com) on 13 May 2026, which provided publicly available competition records and official judges’ scorecards. For each bout, the weight category, competition stage, boxer corner designation, round-level scores awarded by each of the five judges, and judge identities were extracted. Judge identities were linked to the corresponding round-level scores to account for repeated appearances of individual judges across bouts. The extracted data were verified against the official competition records before construction of the analytical dataset.

Under the World Boxing judging system, five judges independently score each round using the 10-Point Must System. Referees and judges are assigned to bouts using the World Boxing-approved Electronic Draw System to ensure neutrality [7]. The official round-level scores assigned by the five judges were used for the inter-judge reliability analyses. Across the 91 bouts contributing to the reliability analyses, 39 individual judges were represented. Individual judges officiated between 1 and 20 bouts (median = 12), and all 91 bouts were evaluated by a unique combination of five judges.

### 2.3. Inter-Judge Reliability Analysis

Inter-judge reliability was assessed using a variance-components approach based on linear mixed-effects models, consistent with generalizability-theory approaches to reliability estimation [24,25,26]. Generalizability theory allows multiple sources of measurement variance, including variance attributable to targets and raters, to be estimated separately and incorporated into reliability estimates [25,26]. This framework is particularly appropriate for complex rater structures in which ratings are not obtained from the same fully crossed set of raters across all targets [24]. Generalizability theory and D-study approaches have also been applied to the evaluation of judging reliability and multi-judge scoring designs in judged sports, including figure skating, rhythmic gymnastics, and taekwondo [27,28,29,30].

Because the composition of the five-judge panel varied across bouts and individual judges could officiate in multiple bouts, judge identity was explicitly incorporated into the model. For each bout-round, each judge’s score was expressed as a signed score margin relative to the red corner, with positive values indicating that the red-corner boxer won the round and negative values indicating that the blue-corner boxer won the round. Thus, 10:9, 10:8, and 10:7 scores in favour of the red corner were coded as +1, +2, and +3, respectively, with corresponding scores in favour of the blue corner coded as −1, −2, and −3. This coding represented each judge’s paired round-level decision as a single observation rather than treating the complementary scores of the two boxers as independent targets. For the variance-components analysis, the resulting signed score margin was treated as a quantitative outcome.

The mixed-effects model was fitted using restricted maximum likelihood (REML) and included random intercepts for bout-round target and judge identity. The observed variance was partitioned into between-target variance ($\sigma_{T}^{2}$), between-judge variance ($\sigma_{J}^{2}$), and residual variance ($\sigma_{e}^{2}$), with the latter representing unexplained disagreement, including judge-by-target variation not captured by the judge random intercept. Variance components were estimated using the lme4 package (version 2.0.6) in R [31]. Following the variance-components definition of absolute reliability, single-judge reliability was calculated as [32]:

(1) $$R_{single}=\frac{\sigma_{T}^{2}}{\sigma_{T}^{2}+\sigma_{J}^{2}+\sigma_{e}^{2}},$$

whereas the reliability of an aggregate measurement based on five judge ratings was calculated as:

(2) $$R_{panel\left(5\right)}=\frac{\sigma_{T}^{2}}{\sigma_{T}^{2}+\left(\sigma_{J}^{2}+\sigma_{e}^{2}\right)/5}$$

Thus, R_single_ represents the absolute reliability of a single judge’s rating, whereas R_panel(5)_ represents the dependability of an aggregate measurement based on five judge ratings. Following generalizability theory/D-study principles, the judge-related and residual error components were divided by 5 to represent the reduction in error variance when 5 ratings contribute to the aggregate measurement [25,26]. D-study approaches examining the reliability of multi-judge scoring designs and the effect of the number of judges have also been applied in judged sports [27,28,29,30]. R_panel(5)_ therefore characterises the dependability of a five-rating measurement design and should not be interpreted as the reliability of the competition’s majority-vote decision rule.

Inter-judge reliability was estimated separately for each round in the overall sample and within each of the three weight categories (60 kg, 75 kg, and 90+ kg). Corresponding 95% confidence intervals were obtained from 5000 parametric bootstrap replications of the fitted mixed-effects models. For descriptive interpretation, reliability coefficients ≥ 0.70 were considered minimally acceptable, whereas coefficients ≥ 0.80 were considered more desirable. These values were treated as descriptive benchmarks rather than universal cut-off points, consistent with previous applications of generalizability theory in sport and sport-related performance assessment [33,34].

### 2.4. Statistical Analysis

Descriptive statistics were used to characterise the analysed sample and the distribution of judges’ scores. Final judges’ decisions (5:0, 4:1, 4:0 and 3:2) were summarised as frequencies and percentages and compared across weight categories using the Fisher–Freeman–Halton exact test, with Cramér’s V as a measure of association. The variance-components reliability models described in Section 2.3 were fitted separately for each round in the overall sample and within each of the three weight categories (60 kg, 75 kg, and 90+ kg). For each model, 95% CIs for both single-judge reliability (R_single_) and five-rating aggregate reliability (R_panel(5)_) were obtained using 5000 parametric bootstrap replications, with the 2.5th and 97.5th percentiles of the bootstrap distributions used as confidence limits [31,35].

To examine differences in reliability across rounds, pairwise comparisons were performed for Round 1 versus Round 2, Round 1 versus Round 3, and Round 2 versus Round 3 using only bouts with complete data for both rounds being compared. This resulted in 91 matched bouts for the Round 1 versus Round 2 comparison and 86 matched bouts for both the Round 1 versus Round 3 and Round 2 versus Round 3 comparisons. Differences in reliability (ΔR) were calculated separately for R_single_ and R_panel(5)_. Sampling uncertainty was evaluated using a non-parametric bout-level bootstrap with 5000 replications [35]. For each replication, matched bouts were resampled with replacement, with all corresponding judge-level ratings and judge identities retained together; the variance-components models were refitted for both rounds, and the difference between the resulting reliability coefficients was calculated. The 2.5th and 97.5th percentiles of the bootstrap distribution of ΔR were used to construct 95% CIs [35]. Two-sided bootstrap-derived *p*-values were calculated for each comparison.

Differences in reliability between weight categories were examined separately within each round. Pairwise comparisons were performed for 60 kg versus 75 kg, 60 kg versus 90+ kg, and 75 kg versus 90+ kg. Within each bootstrap replication, bouts were independently resampled with replacement within each weight category, while retaining all judge-level ratings and judge identities for each bout. The variance-components models were then refitted, and pairwise differences in R_single_ and R_panel(5)_ were calculated. This procedure was repeated 5000 times, and 95% percentile CIs and two-sided bootstrap-derived *p*-values were obtained for each ΔR [35].

To control the family-wise error rate, Holm adjustment [36] was applied separately to the three between-round comparisons within the overall sample and within each weight category (60 kg, 75 kg, and 90+ kg). Between-category comparisons were treated as secondary analyses, and Holm adjustment was applied separately to the three pairwise category comparisons within each round. The reported bootstrap CIs were unadjusted percentile intervals; multiplicity adjustment was applied only to the *p*-values. Statistical significance was assessed using Holm-adjusted *p*-values, with *p* < 0.05 considered statistically significant. A 95% CI for ΔR including zero was interpreted as being compatible with no difference in reliability; non-significant results were not interpreted as evidence of equivalence.

Because advancing boxers could contribute observations from more than one bout, a sensitivity analysis was conducted to assess whether repeated participation by boxers materially affected the reliability estimates. In the 91-bout reliability dataset, 95 unique boxers were represented, of whom 50 appeared in more than one bout. A total of 1000 random subsets were generated in which no boxer contributed more than one bout within a given subset. R_single_ and R_panel(5)_ were recalculated separately for each round within each subset, and the median and 2.5th–97.5th percentile range of the resulting reliability estimates were compared with the corresponding primary estimates. These percentile ranges describe variation across the generated subsets and were not interpreted as confidence intervals. The results of this sensitivity analysis are reported in Supplementary Table S1.

Because the study constituted a retrospective census of all eligible bouts across the three selected weight categories, no conventional a priori sample size or power calculation was performed. Statistical uncertainty was instead quantified using confidence intervals around the reliability estimates and between-group differences, and non-significant findings were not interpreted as evidence of equivalence.

Statistical analyses were performed using IBM SPSS Statistics, version 31 (IBM Corp., Armonk, NY, USA), and R, version 4.6.0 (R Foundation for Statistical Computing, Vienna, Austria). The variance-component reliability models and bootstrap analyses were conducted in R using the lme4 package (version 2.0.6) [31], with the dplyr package (version 1.2.1) used for data processing [37]. Random seeds were set for all bootstrap analyses to ensure computational reproducibility. The anonymised analytical dataset and complete R code for the reliability analyses, including the exact random seeds and analysis procedures, are available in the Supplementary Materials. Statistical significance was set at *p* < 0.05 where applicable.

## 3. Results

The distribution of judges’ round scores by round and weight category is presented in Table 1. Of the 1340 valid judge-round decisions, 1316 (98.2%) were scored 10:9, while only 24 (1.8%) were scored 10:8; no 10:7 scores were recorded. This pattern was consistent across rounds, with 10:9 scores accounting for 97.1–99.1% of all decisions. Across weight categories, 10:8 scores were most frequent in the 60 kg category (2.4%), followed by the 90+ kg category (1.8%) and the 75 kg category (0.6%).

The distribution of final judges’ decisions, both overall and across the three weight categories, is presented in Table 2. Four bouts that were decided by knockout, referee stoppage, or disqualification rather than by the judges’ final scores were excluded from this analysis. Of the 90 bouts decided by judges’ scores, 62 (68.9%) resulted in a unanimous 5:0 decision, while 14 (15.6%) were decided by a 4:1 margin, one (1.1%) by a 4:0 margin, and 13 (14.4%) by a 3:2 margin. Unanimous decisions were most frequent in the 60 kg and 75 kg categories (71.4% each), followed by the 90+ kg category (63.0%). The distribution of final judges’ decisions (5:0, 4:1, 4:0, and 3:2) did not differ significantly across weight categories (Fisher–Freeman–Halton exact test, *p* = 0.931), with a small association between weight category and decision type (Cramér’s V = 0.124).

Table 3 presents the reliability coefficients for individual judges (R_single_) and for an aggregate measurement based on five judges (R_panel(5)_) across rounds and weight categories. Overall, reliability was consistently higher for the five-rating aggregate than for individual judge ratings in every round and weight category. Across all bouts, R_single_ was 0.617 (95% CI: 0.521–0.693) in Round 1, 0.634 (95% CI: 0.539–0.709) in Round 2, and 0.687 (95% CI: 0.597–0.754) in Round 3. In contrast, the corresponding five-rating aggregate reliability coefficients remained consistently high, ranging from 0.889 (95% CI: 0.845–0.919) in Round 1 to 0.916 (95% CI: 0.881–0.939) in Round 3. Within the 60 kg category, single-judge reliability ranged from 0.604 to 0.719, while five-rating aggregate reliability ranged from 0.884 to 0.927 across rounds. The Round 3 R_panel(5)_ estimate of 0.927 (95% CI: 0.874–0.955) was the highest category-specific estimate observed across the study. In the 75 kg category, single-judge reliability showed greater variability, increasing from 0.504 (95% CI: 0.274–0.672) in Round 1 to 0.666 (95% CI: 0.452–0.795) in Round 2 before declining slightly to 0.597 (95% CI: 0.360–0.748) in Round 3. Nevertheless, five-rating aggregate reliability remained high throughout (0.836–0.909). Similarly, in the 90+ kg category, R_single_ ranged from 0.599 to 0.685, whereas R_panel(5)_ ranged from 0.882 to 0.916. Numerically, Round 3 produced the highest reliability estimates within this category.

Table 4 presents pairwise comparisons of single-judge and five-rating aggregate reliability between rounds in the overall sample and within each weight category. In the overall sample, no statistically significant differences in reliability were detected between Round 1 and Round 2, Round 1 and Round 3, or Round 2 and Round 3 after Holm adjustment (all p^Ha^ ≥ 0.356). The largest numerical difference was observed between Rounds 1 and 3 for single-judge reliability (ΔR_single_ = −0.073, 95% CI: −0.177 to 0.037), with the corresponding difference for the five-rating aggregate being smaller (ΔR_panel(5)_ = −0.028, 95% CI: −0.074 to 0.014). Similarly, no statistically significant between-round differences were detected within the 60 kg, 75 kg, or 90+ kg categories (all p^Ha^ ≥ 0.398). Across all category-specific comparisons, the 95% confidence intervals for both ΔR_single_ and ΔR_panel(5)_ included zero. These results indicate that no statistically significant between-round differences in either single-judge or five-rating aggregate reliability were detected, either overall or within individual weight categories.

Table 5 presents pairwise comparisons of single-judge and five-rating aggregate reliability between weight categories within each round. No statistically significant differences in reliability were detected between weight categories in any round after Holm adjustment (all p^Ha^ ≥ 0.594). In Round 1, the largest numerical difference was observed between the 60 kg and 75 kg categories (ΔR_single_ = 0.170, 95% CI: −0.090 to 0.410; ΔR_panel(5)_ = 0.076, 95% CI: −0.037 to 0.234). In Round 2, differences between categories were small, with the 75 kg and 90+ kg categories showing nearly identical reliability estimates (ΔR_single_ = 0.002, 95% CI: −0.251 to 0.250; ΔR_panel(5)_ = 0.001, 95% CI: −0.109 to 0.100). In Round 3, the largest numerical difference was again observed between the 60 kg and 75 kg categories (ΔR_single_ = 0.122, 95% CI: −0.090 to 0.369; ΔR_panel(5)_ = 0.046, 95% CI: −0.032 to 0.176). Across all nine comparisons, the 95% confidence intervals for both ΔR_single_ and ΔR_panel(5)_ included zero, providing no statistically significant evidence of between-category differences in either single-judge or five-rating aggregate reliability.

## 4. Discussion

This study examined inter-judge reliability under the World Boxing 10-Point Must Scoring System using official scores from 94 men’s bouts at the 2025 World Boxing Championships. A clear finding was that reliability was consistently higher when the ratings of five judges were aggregated than when individual judge ratings were considered. Across all bouts, single-judge reliability ranged from 0.617 to 0.687, compared with 0.889 to 0.916 for the five-rating aggregate. Some numerical variation was observed between rounds, but none of the between-round comparisons reached statistical significance after adjustment for multiple comparisons. Likewise, no statistically significant differences were detected between weight categories in any round. Judges also made extensive use of the 10:9 score, which accounted for 98.2% of valid judge-round decisions, compared with only 1.8% scored 10:8; no 10:7 scores were recorded. Of the 90 bouts decided on the judges’ scorecards, 68.9% ended in unanimous 5:0 decisions, while the distribution of 5:0, 4:1, 4:0, and 3:2 decisions was similar across weight categories. Taken together, these findings highlight the difference between an individual judge’s reliability and the reliability achieved when the ratings of five judges are combined, with no statistically significant evidence of systematic differences across rounds or weight categories.

This distinction between individual and aggregated ratings is important when interpreting the present findings. Although single-judge reliability was lower, ranging from moderate to relatively high across the different analyses, combining five ratings produced considerably higher reliability estimates. At the category level, R_panel(5)_ ranged from 0.836 to 0.927, showing a similar pattern to that observed in the full sample. From a measurement perspective, this is expected because aggregating several independent ratings reduces the influence of judge-specific and residual disagreement [29,30]. However, R_panel(5)_ does not represent the reliability of the majority-vote bout decision. Rather, it reflects the reliability of an aggregate measurement based on five judge ratings under the fitted variance-component model. This distinction is particularly relevant in World Boxing, where five judges independently evaluate each bout. The present results therefore show that disagreement remains meaningful at the level of an individual judge, while considerably greater reliability is achieved when the five ratings are considered together.

### 4.1. Inter-Judge Reliability in Judged and Combat Sports

The five-rating aggregate reliability observed in the present study was broadly comparable to the relatively high levels of interrater reliability reported in other judged sports. Braeunig [14], for example, found generally high interrater reliability across Olympic winter sports, although the magnitude varied across disciplines. Average ICC values were approximately 0.857 for individual elements in figure skating, 0.895 for ski jumping, and 0.990 for snowboarding. However, numerical comparisons across studies should be interpreted with caution because reliability estimates depend on the statistical model and definition used, the number of judges or ratings aggregated, and the variability of the underlying scores [28]. Moreover, the variance-component coefficients used in the present study are not directly equivalent to the ICC estimates reported in these earlier studies. One of the broader conclusions from that work was that reliability depends not only on judge expertise but also on how a scoring system is structured and how clearly its criteria are defined. The current findings fit reasonably well within that perspective. Despite the speed and complexity of elite boxing, aggregating the five judge ratings produced consistently high reliability estimates.

Previous studies applying generalizability theory in judged sports provide further context for the distinction between single-judge and five-rating aggregate reliability observed in the present study. In figure skating, Lee et al. [29] reported that the generalizability coefficient increased from 0.865 when performance was evaluated by a single judge to 0.928 with two judges and 0.970 with five judges, illustrating the expected gain in dependability when several ratings are combined. A similar pattern was observed in taekwondo poomsae, where Cho [30] found substantially lower coefficients for a single rater than for measurement designs based on several raters; for example, the coefficient for the accuracy component increased from 0.348 with one judge to 0.728 with five judges, while the corresponding values for expressivity increased from 0.399 to 0.769. The present findings extend this pattern to elite international boxing. Across the three rounds, R_single_ ranged from 0.617 to 0.687, whereas R_panel(5)_ ranged from 0.889 to 0.916, indicating considerably greater measurement reliability when five judge ratings were aggregated. These coefficients should not be interpreted as directly equivalent across studies because the scoring scales, objects of measurement, measurement designs, and definitions of error differ. Nevertheless, the common pattern is consistent with the generalizability theory principle that aggregating multiple ratings reduces the contribution of rater-related and residual error to the aggregate measurement. Huang and Foote [27] similarly identified rater-related variance as a relevant source of scoring variability in figure skating despite high overall generalizability coefficients. Thus, the high R_panel(5)_ observed in the present study should not be interpreted as evidence that individual-level disagreement is negligible; rather, the lower R_single_ estimates indicate that individual-judge consistency remains an important target for judge education and evaluation.

Research into combat sports also shows that such agreement cannot simply be taken for granted. Myers et al. [12] highlighted that judging consistency in Muay Thai depended substantially on how scoring criteria were understood and applied. Thai officials were more consistent than UK-trained officials, while the UK judges became more consistent when they used the more explicitly defined Thai criteria. The authors argued that clearer operationalisation of the scoring criteria was an important part of this improvement. This has a clear parallel with World Boxing, where judges must integrate several aspects of performance when determining the winner of a round.

In the present study, the consistently high R_panel(5)_ estimates indicate that considerably greater reliability was achieved when the five judge ratings were aggregated than when a single judge rating was considered. At the same time, the lower R_single_ estimates show that meaningful disagreement remained at the individual-judge level. However, because panel composition varied across bouts, R_panel(5)_ should not be interpreted as the reliability of a particular five-judge panel or of the majority-vote bout decision. Nor do the present findings show that the 10-Point Must System itself caused the observed reliability, as no alternative scoring system was examined.

The contrast with kickboxing is particularly interesting. Hoelbling et al. [38] reported low agreement among judges at the 2021 WAKO World Championships. Their findings also highlighted how demanding combat-sport judging can be from a perceptual and cognitive perspective: officials must detect, classify, remember, and evaluate technical actions that often occur in rapid succession. Direct comparison of their κ coefficients with the reliability coefficients reported in the present study would not be appropriate because the statistics and underlying data structures differ. Even so, the broader comparison is useful. Elite competition and experienced judges do not automatically guarantee high agreement, which makes the relatively high reliability of the five-rating aggregate in the present World Boxing sample notable.

Boxing places similar demands on its judges. Offensive and defensive actions occur quickly, and judges must decide not only whether the actions were successful but also how they contribute to one boxer’s overall superiority in a round. Avugos et al. [39] identified cognitive load, time pressure, expertise, and contextual information as relevant factors in sports-officiating decisions. Against this background, the difference observed between single-judge and five-rating aggregate reliability is particularly relevant. Although individual judge ratings showed appreciable disagreement, aggregating five ratings substantially reduced their influence on the resulting measurement. This suggests that the multi-judge structure used in World Boxing may provide an important measurement advantage in a demanding officiating environment.

### 4.2. Scoring Distribution and the Meaning of High Reliability

The scoring distribution provides useful context for the reliability findings. Of 1340 valid judge-round decisions, 1316 (98.2%) were scored 10:9, only 24 (1.8%) were scored 10:8, and none were scored 10:7. The present reliability estimates therefore mainly reflect judging within a competition dominated by relatively narrow 10:9 decisions.

This should be considered when generalising the findings. Because reliability estimates are influenced by the distribution and between-target variability of the observed scores, these estimates cannot automatically be extended to competitions with a larger proportion of 10:8 or 10:7 rounds. The results indicate relatively high aggregate reliability for five-rating scores under the scoring conditions observed at this World Championship, but the predominance of 10:9 scores should not be interpreted as evidence of judging consistency. Rather, it describes the scoring context in which the reliability estimates were obtained. The predominance of 10:9 scores may also reflect the multidimensional nature of boxing judging. Dunn et al. [11] showed that judges’ perceptions of dominance were not explained by punch volume alone. Punching accuracy and movement characteristics helped distinguish winners from losers, and the combined model correctly classified 84.6% of bout outcomes. Judges therefore appear to integrate several performance cues when deciding round superiority.

The distribution of final bout decisions provides complementary information to the round-level reliability analysis. Of the 90 bouts decided by judges’ scores, 62 (68.9%) ended in unanimous 5:0 decisions, while 14 (15.6%) ended 4:1, one (1.1%) ended 4:0, and 13 (14.4%) ended 3:2. Thus, although the five-rating aggregate showed relatively high reliability, complete unanimity in the final bout decision was not always reached. This is not inconsistent with the reliability findings, as the two analyses address different aspects of judging: R_single_ and R_panel(5)_ quantify the reliability of individual and aggregated round-level judge ratings, respectively, whereas the final decision distribution reflects the degree of unanimity in the overall bout outcome.

The distribution of final decisions was also similar across weight categories (*p* = 0.931, Cramér’s V = 0.124), with no statistically significant evidence that the distribution of final decisions differed among the three weight categories.

### 4.3. Reliability Across Rounds

Reliability remained broadly comparable across the three rounds. In the full sample, R_single_ was 0.617 in Round 1, 0.634 in Round 2, and 0.687 in Round 3, while R_panel(5)_ was 0.889, 0.897, and 0.916, respectively. Although both coefficients were numerically highest in Round 3, no statistically significant between-round differences were detected after adjustment for multiple comparisons. The same was true within each weight category. These numerical patterns should therefore not be interpreted as evidence of a systematic round effect. Importantly, there was also no indication of a progressive decline in reliability from Round 1 to Round 3. This is relevant because judging a boxing bout requires sustained attention under considerable perceptual and cognitive demands. Bloß et al. [40] reported mixed evidence regarding the relationship between officiating load and decision-making performance, highlighting the need for caution when attributing changes in judging performance to fatigue alone.

Several factors could plausibly influence judging conditions across rounds, including changes in pacing, tactical behaviour, action density, and the clarity of performance differences between opponents. Previous research has shown that boxing has a complex intermittent temporal structure and that technical–tactical and activity patterns may change over the course of a bout [9,11,41,42]. Such changes could theoretically affect the cues available to judges when evaluating round superiority. However, the present study did not assess fatigue, score status, action density, or technical–tactical behaviour, and no statistically significant between-round differences in reliability were detected. Any explanation of the observed numerical variation in terms of these mechanisms would therefore be speculative. Future studies combining judge-level scoring data with round-specific technical–tactical indicators may help determine whether particular phases of a bout create more challenging judging conditions.

### 4.4. Weight-Category-Specific Patterns

Weight category was examined because boxing behaviour differs across the competitive weight spectrum. Gutiérrez-Santiago et al. [9], for example, identified differences in temporal and technical–tactical characteristics between Olympic flyweight, middleweight, and super-heavyweight bouts. Differences in pace, movement, fighting distance, and action frequency could theoretically alter the demands placed on judges. Comparable weight-category-specific patterns have been reported in other combat sports. In international taekwondo, weight category was associated with differences in fighting time, preparatory time, and the number of exchanges, whereas world-class judo athletes showed category-specific differences in gripping configurations and attack systems. Collectively, these findings support the broader premise that weight category can shape the temporal and technical–tactical information available to officials, although they do not establish that some categories are inherently easier to judge [19,43].

The current findings provide little evidence of a systematic weight-category effect. None of the nine direct comparisons between weight categories reached statistical significance. Although some numerical variation was observed, five-rating aggregate reliability remained relatively high across all three categories and rounds, ranging from 0.836 to 0.927. Single-judge reliability showed greater variation, but the between-category comparisons likewise provided no statistically significant evidence of systematic differences. All between-category comparisons also remained non-significant after Holm adjustment. Thus, although some descriptive variation in reliability was present across weight categories, the bootstrap analyses provided no statistically significant evidence that these differences exceeded what would be expected from sampling variation. The findings, therefore, do not indicate that any of the three examined weight categories were consistently associated with higher or lower judging reliability. Despite previously reported weight-category differences in the temporal and technical–tactical characteristics of combat sports, these differences were not accompanied by statistically significant differences in inter-judge reliability in the present sample.

Similarly, no statistically significant between-round differences were detected within any of the three weight categories after Holm adjustment. Although activity patterns and temporal structure may differ across weight categories [9], these characteristics were not examined in the present study. The observed numerical variation across rounds should therefore be interpreted cautiously and not as evidence of weight-category-specific differences in judging reliability.

### 4.5. Reliability, Accuracy, and Judging Bias

High reliability does not necessarily mean accurate or unbiased judging. Reliability tells us whether judges score the same performance similarly; it does not tell us whether their common evaluation is objectively correct. Heiniger and Mercier [44] demonstrated this distinction in international gymnastics, where meaningful differences in individual judge accuracy and evidence of national bias were identified despite an established judging framework. Similarly, Balmer et al. [45] reported evidence of home advantage in European Championship boxing, particularly when outcomes depended on judges rather than an objective stoppage. More recently, Baumann and Singleton [1] proposed a consensus-based scoring approach intended to reduce the influence of a potentially partisan individual judge on close boxing outcomes.

The current results do not contradict this literature. They show that aggregating five judges’ ratings yielded relatively high reliability at the 2025 World Championships, but this does not establish that the underlying ratings were accurate or free of bias. The distinction between R_single_ and R_panel(5)_ is particularly relevant here: the higher reliability of the five-rating aggregate indicates that combining ratings reduces the influence of individual-level disagreement, but it cannot determine whether judges share a systematic bias or whether their evaluations correspond to an external criterion of accuracy. Future judge evaluation should therefore combine reliability assessment with individual longitudinal monitoring. Judges who repeatedly diverge from other judges could be identified for further review, although disagreement itself should not automatically be treated as error. Independent expert assessment or another defensible external criterion would be required to distinguish disagreement from actual inaccuracy. Such longitudinal monitoring could consider not only the magnitude of a judge’s deviation from other judges, but also whether such deviations form a systematic pattern associated with particular boxers, nationalities, or competitive contexts.

### 4.6. Practical Implications and Future Directions in Judging

For World Boxing, the relatively high reliability achieved by aggregating five judge ratings is encouraging, although the lower single-judge reliability indicates that disagreement at the individual level remains relevant. The substantial difference between single-judge and five-rating aggregate reliability also identifies a clear target for further development of the judging system. Although aggregation effectively reduces the influence of individual-level disagreement, continued judge education and evaluation should aim to improve the consistency of individual ratings, thereby reducing the extent to which reliable measurement depends on aggregation across multiple judges [29,30]. Particular attention could be directed towards rounds in which individual judges repeatedly diverge from the other ratings, using these situations as standardised material for targeted training and feedback.

For coaches and athletes, the findings reinforce the practical importance of making scoring-relevant superiority sufficiently clear for judges viewing the bout from different positions to recognise it. This is not simply a matter of being more active, as punching accuracy, effective actions, and movement characteristics also appear to shape judges’ perceptions of dominance [11]. The present findings are also encouraging in the context of World Boxing’s recent efforts to standardise and strengthen referee and judge certification [5]. Although the current study cannot determine whether these initiatives directly contributed to the observed reliability, the results provide an empirical benchmark for individual-judge and five-rating aggregate reliability under the current judging framework at the highest level of World Boxing competition.

Technology could play a useful role in this process. Hoelbling et al. [38] demonstrated how JudgED, a serious-game environment based on standardised video scenarios, can support judge training in combat sports. A similar approach could be developed for boxing using close 10:9 rounds, complex exchanges, and other situations that are difficult to judge consistently. AI-assisted analysis may offer another avenue. Zhang et al. [46] reported strong agreement between AI-generated and international referee decisions in 241 video-review cases from Olympic taekwondo (κ = 0.897), while duBoef et al. [47] used computer-vision data to model judging decisions in professional boxing. Although these approaches are not directly transferable to World Boxing, they show how technology could complement human judging through training, monitoring, and post-competition review rather than replace judges.

However, several limitations should be acknowledged. Only men’s bouts from three weight categories were analysed, and sample sizes differed across weight categories and, for round-specific analyses, according to the number of rounds completed. The findings therefore cannot automatically be generalised to women’s boxing, other weight categories, or different competition levels. Judging-panel composition also varied between bouts. This reflects actual competition practice and was addressed analytically by modelling judge identity as a random effect. Nevertheless, the available data contained only one observation for each judge-by-bout-round combination, so unexplained judge-by-target variation could not be estimated as a separate variance component and instead remained part of the residual variance. Some boxers also appeared in more than one bout as they progressed through the competition. Sensitivity analyses based on independent-bout subsets produced estimates similar to the primary analysis, suggesting that repeated boxer appearances did not materially influence the reliability estimates, although this dependence remains a feature of the competition structure. The signed score margin was treated as a quantitative outcome in the linear mixed-effects models. This provided a practical way to preserve the direction and magnitude of judge disagreement while avoiding treating complementary red- and blue-corner scores as independent observations, but the outcome remained discrete and had a limited range. The study assessed reliability rather than criterion validity. Without an independent gold-standard assessment, high reliability cannot establish that judges were objectively correct. Judge nationality, experience, position, certification history, and workload were also not examined, despite evidence that individual and contextual factors can influence officiating [39,44]. Finally, technical–tactical characteristics were not linked directly to judging reliability. Consequently, the numerical variation observed across rounds and weight categories cannot be attributed to differences in fatigue, pacing, tactical behaviour, or other competition characteristics. The rarity of 10:8 scores and absence of 10:7 scores also mean that the study provides much stronger evidence about reliability in closely scored rounds than in clearly one-sided rounds.

Future research should include both sexes, all weight categories, and multiple competitions, while also following individual judges over time. Linking official scores with judge characteristics, independent expert assessments, and technical–tactical data could help explain not only when judges disagree, but why, providing a more complete picture of reliability, accuracy, and potential bias. Psychophysiological factors should also be considered, particularly given evidence of variation in sleep quality among combat-sport referees [48]. Examining whether sleep and fatigue are associated with judging consistency represents a relevant direction for future research.

Judge training should also be evaluated experimentally, with future intervention studies examining whether targeted training can improve single-judge reliability and reduce individual-level disagreement. In particular, comparing R_single_ and R_panel(5)_ before and after standardised training could help determine whether improvements occur at the individual-judge level and whether improvements at the individual-judge level reduce the extent to which aggregation compensates for individual-level disagreement. Video-based and serious-game approaches, such as JudgED, could provide a practical way to expose judges to difficult yet standardised situations [38]. Emerging AI-assisted methods could further complement this process through judge training, monitoring, and quality control [46,47]. At present, however, these technologies appear more appropriate as decision-support tools than as replacements for trained officials.

## 5. Conclusions

Judging at the 2025 World Boxing Championships showed a clear difference between the reliability of individual judge ratings and that of an aggregate measurement based on five judge ratings. Single-judge reliability was moderate to relatively high, whereas five-rating aggregate reliability was consistently high across the three rounds and weight categories examined. This distinction provides an important empirical benchmark for the current World Boxing judging framework: although aggregating five ratings substantially reduces the influence of individual-level disagreement, the lower single-judge reliability underscores the need to identify individual judging consistency as an important target for further education, evaluation, and training. Although some numerical variation in reliability was observed across rounds, none of the between-round differences was statistically significant after adjustment for multiple comparisons. Similarly, no statistically significant differences in reliability were detected between weight categories in any round. These findings provide no statistically significant evidence of systematic round- or weight-category-specific differences in judging reliability within the categories examined. The predominance of 10:9 scores characterised the scoring context of the competition, while the high proportion of unanimous 5:0 bout decisions provided complementary information on agreement in final bout outcomes. Neither should be interpreted as direct evidence of reliability, and high reliability should not be taken as evidence of accuracy or of the absence of bias. Accordingly, the present findings inform the reproducibility of official judge ratings and the measurement value of aggregating multiple ratings, but they cannot establish whether the scores were correct, appropriately aligned with the regulatory criteria, or free from systematic bias. Future research that combines round-level scoring with individual judges’ characteristics, technical–tactical analysis, and independent expert assessment is needed to better understand the sources of judging disagreement and to support the development of evidence-based judge training and decision-support systems. Intervention studies should also determine whether targeted judge training can improve single-judge reliability and reduce individual-level disagreement.

## Figures and Tables

**Table 1:** Distribution of judges’ round scores by round and weight category.

Round	Weight Category	Judge-Round Decisions, *n*	10:9, *n* (%)	10:8, *n* (%)	10:7, *n* (%)
Round 1	60 kg	210	200 (95.2)	10 (4.8)	0 (0.0)
	75 kg	110	110 (100.0)	0 (0.0)	0 (0.0)
	90+ kg	135	132 (97.8)	3 (2.2)	0 (0.0)
	Overall	455	442 (97.1)	13 (2.9)	0 (0.0)
Round 2	60 kg	210	209 (99.5)	1 (0.5)	0 (0.0)
	75 kg	110	109 (99.1)	1 (0.9)	0 (0.0)
	90+ kg	135	133 (98.5)	2 (1.5)	0 (0.0)
	Overall	455	451 (99.1)	4 (0.9)	0 (0.0)
Round 3	60 kg	200	196 (98.0)	4 (2.0)	0 (0.0)
	75 kg	105	104 (99.0)	1 (1.0)	0 (0.0)
	90+ kg	125	123 (98.4)	2 (1.6)	0 (0.0)
	Overall	430	423 (98.4)	7 (1.6)	0 (0.0)
All rounds	60 kg	620	605 (97.6)	15 (2.4)	0 (0.0)
	75 kg	325	323 (99.4)	2 (0.6)	0 (0.0)
	90+ kg	395	388 (98.2)	7 (1.8)	0 (0.0)
	Overall	1340	1316 (98.2)	24 (1.8)	0 (0.0)

**Table 2:** Distribution of final judges’ decisions overall and by weight category.

Weight Category	Bouts, *n*	5:0, *n* (%)	4:1, *n* (%)	4:0, *n* (%)	3:2, *n* (%)
60 kg	42	30 (71.4)	6 (14.3)	0 (0.0)	6 (14.3)
75 kg	21	15 (71.4)	3 (14.3)	0 (0.0)	3 (14.3)
90+ kg	27	17 (63.0)	5 (18.5)	1 (3.7)	4 (14.8)
Overall	90	62 (68.9)	14 (15.6)	1 (1.1)	13 (14.4)

Note. Values are presented as *n* (%). A 5:0 result represents a unanimous decision, whereas 4:1, 4:0, and 3:2 results represent non-unanimous decisions. The 4:0 result occurred in a single bout in which four judges favoured the winning boxer and one judge had a tied scorecard.

**Table 3:** Single-Judge and Aggregate Reliability Based on Five Judge Ratings Across Rounds and Weight Categories.

Round	Weight Category	*n*	R_single_	95% CI	R_panel(5)_	95% CI
Round 1	Overall	91	0.617	0.521–0.693	0.889	0.845–0.919
	60 kg	42	0.674	0.538–0.769	0.912	0.853–0.943
	75 kg	22	0.504	0.274–0.672	0.836	0.654–0.911
	90+ kg	27	0.599	0.394–0.731	0.882	0.765–0.931
Round 2	Overall	91	0.634	0.539–0.709	0.897	0.854–0.924
	60 kg	42	0.604	0.448–0.713	0.884	0.802–0.925
	75 kg	22	0.666	0.452–0.795	0.909	0.805–0.951
	90+ kg	27	0.664	0.476–0.779	0.908	0.820–0.946
Round 3	Overall	86	0.687	0.597–0.754	0.916	0.881–0.939
	60 kg	40	0.719	0.580–0.808	0.927	0.874–0.955
	75 kg	21	0.597	0.360–0.748	0.881	0.738–0.937
	90+ kg	25	0.685	0.498–0.799	0.916	0.832–0.952

Note. R_single_ = absolute reliability of a single judge’s rating; R_panel(5)_ = dependability of an aggregate measurement based on five judge ratings; CI = confidence interval. Confidence intervals were obtained from 5000 parametric bootstrap replications. R_panel(5)_ characterises the dependability of the five-rating measurement design and does not represent the reliability of the competition’s majority-vote decision rule.

**Table 4:** Pairwise comparisons of single-judge and five-rating aggregate reliability between rounds in the overall sample and by weight category.

Sample/Weight Category	Comparison	Matched *n*	ΔR_single_	95% CI	ΔR_panel(5)_	95% CI	p^U^	p^Ha^
Overall	R1 vs. R2	91	−0.018	−0.139 to 0.103	−0.007	−0.058 to 0.042	0.789	0.789
	R1 vs. R3	86	−0.073	−0.177 to 0.037	−0.028	−0.074 to 0.014	0.192	0.384
	R2 vs. R3	86	−0.073	−0.161 to 0.018	−0.028	−0.066 to 0.007	0.119	0.356
60 kg	R1 vs. R2	42	0.070	−0.123 to 0.242	0.028	−0.050 to 0.102	0.482	0.763
	R1 vs. R3	40	−0.058	−0.190 to 0.075	−0.020	−0.075 to 0.027	0.382	0.763
	R2 vs. R3	40	−0.130	−0.285 to 0.035	−0.050	−0.123 to 0.013	0.133	0.398
75 kg	R1 vs. R2	22	−0.162	−0.368 to 0.095	−0.073	−0.208 to 0.044	0.238	0.715
	R1 vs. R3	21	−0.078	−0.295 to 0.152	−0.037	−0.175 to 0.079	0.557	0.791
	R2 vs. R3	21	0.053	−0.064 to 0.162	0.022	−0.030 to 0.081	0.396	0.791
+90 kg	R1 vs. R2	27	−0.065	−0.276 to 0.127	−0.026	−0.129 to 0.055	0.507	1.000
	R1 vs. R3	25	−0.077	−0.326 to 0.145	−0.030	−0.150 to 0.061	0.431	1.000
	R2 vs. R3	25	−0.042	−0.199 to 0.097	−0.016	−0.089 to 0.039	0.453	1.000

Note. ΔR = reliability in the first round minus reliability in the second round of each comparison; R_single_ = absolute reliability of a single judge’s rating; R_panel(5)_ = dependability of an aggregate measurement based on five judge ratings; CI = confidence interval; p^U^ = unadjusted two-sided bootstrap-derived *p*-value; p^Ha^ = Holm-adjusted *p*-value. Negative ΔR values indicate higher reliability in the second round of the comparison. Confidence intervals are unadjusted percentile intervals obtained from 5000 non-parametric bout-level bootstrap replications. The Holm adjustment was applied separately to the three between-round comparisons within the overall sample and within each weight category. The bootstrap *p*-values for ΔR_single_ and ΔR_panel(5)_ were identical because R_panel(5)_ is a strictly increasing transformation of R_single_; therefore, a single unadjusted and a single Holm-adjusted *p*-value are reported for each comparison.

**Table 5:** Pairwise Comparisons of Single-Judge and Five-Rating Aggregate Reliability Between Weight Categories Within Each Round.

Round	Comparison	ΔR_single_	95% CI	ΔR_panel(5)_	95% CI	p^U^	p^Ha^
R1	60 vs. 75 kg	0.170	−0.090 to 0.410	0.076	−0.037 to 0.234	0.198	0.594
	60 vs. 90+ kg	0.075	−0.120 to 0.316	0.030	−0.051 to 0.149	0.394	0.788
	75 vs. 90+ kg	−0.094	−0.335 to 0.191	−0.046	−0.200 to 0.096	0.592	0.788
R2	60 vs. 75 kg	−0.062	−0.281 to 0.196	−0.025	−0.112 to 0.091	0.675	1.000
	60 vs. 90+ kg	−0.060	−0.253 to 0.175	−0.024	−0.106 to 0.078	0.684	1.000
	75 vs. 90+ kg	0.002	−0.251 to 0.250	0.001	−0.109 to 0.100	0.964	1.000
R3	60 vs. 75 kg	0.122	−0.090 to 0.369	0.046	−0.032 to 0.176	0.257	0.770
	60 vs. 90+ kg	0.034	−0.176 to 0.252	0.012	−0.062 to 0.100	0.692	0.936
	75 vs. 90+ kg	−0.088	−0.360 to 0.163	−0.035	−0.174 to 0.068	0.468	0.936

Note. ΔR = reliability in the first weight category minus reliability in the second weight category of each comparison; R_single_ = absolute reliability of a single judge’s rating; R_panel(5)_ = dependability of an aggregate measurement based on five judge ratings; CI = confidence interval; p^U^ = unadjusted two-sided bootstrap-derived *p*-value; p^Ha^ = Holm-adjusted *p*-value. Positive ΔR values indicate higher reliability in the first weight category, and negative values indicate higher reliability in the second weight category. Confidence intervals are unadjusted percentile intervals obtained from 5000 non-parametric bout-level bootstrap replications. The Holm adjustment was applied separately to the three between-category comparisons within each round. The bootstrap *p*-values for ΔR_single_ and ΔR_panel(5)_ were identical because R_panel(5)_ is a strictly increasing transformation of R_single_; therefore, a single unadjusted and a single Holm-adjusted *p*-value are reported for each comparison.

## Data Availability

The original competition records and official judges’ scorecards are publicly available from the official 2025 World Boxing Championships records. The processed analytical dataset and R code used to reproduce the reliability analyses and reported tables are provided in the Supplementary Materials.

## Supplementary Materials

The following supporting information can be downloaded at: https://www.mdpi.com/article/10.3390/sports14090403/s1, Table S1: Sensitivity analysis excluding repeated boxer appearances; File S1: R_Code_World_Boxing_2025; File S2: World_Boxing_2025_analytic_dataset.

## Abbreviations

The following abbreviations are used in this manuscript:

AI	Artificial Intelligence
CI	Confidence Interval
LMM	Linear Mixed-Effects Model
REML	Restricted Maximum Likelihood
R_single_	Single-Judge Reliability
R_panel(5)_	Reliability of an Aggregate Measurement Based on Five Judge Ratings
ΔR	Difference in Reliability Coefficients
