# Supplementary material for: Consistency of Judging Under the World Boxing 10-Point Must Scoring System: Single-Judge and Five-Judge Aggregate Reliability Across the 60 kg, 75 kg, and 90+ kg Categories at the 2025 World Boxing Championships
Source: Sports (Basel). 2026 Sep 14;14(9):403. doi: 10.3390/sports14090403 (PMC13611802; doi:10.3390/sports14090403)
Supplement: Supplementary file 1 [file sports-14-00403-s001.zip › Supplementary Table S1.pdf]

Supplementary Table S1. Sensitivity analysis excluding repeated boxer appearances.

Round	Primary R_{single}	Sensitivity R_{single}, median (2.5th–97.5th percentile)	Primary $R_{panel(5)}$	Sensitivity $R_{panel(5)}$, median (2.5th–97.5th percentile)
Round 1	0.617	0.648 (0.534–0.752)	0.889	0.902 (0.851–0.938)
Round 2	0.634	0.626 (0.527–0.726)	0.897	0.893 (0.848–0.930)
Round 3	0.687	0.686 (0.593–0.785)	0.916	0.916 (0.879–0.948)

Note. The sensitivity analysis was based on 1,000 randomly generated independent-bout subsets in which no boxer appeared more than once within each subset. Values for the sensitivity analyses are presented as the median and 2.5th–97.5th percentile across iterations. R_{single} = single-judge reliability; $R_{panel(5)}$ = reliability of an aggregate measurement based on five judge ratings.

Sensitivity analyses restricted to randomly generated independent-bout subsets yielded reliability estimates closely comparable to those of the primary analysis across all three rounds (Supplementary Table S1), indicating that repeated boxer appearances did not materially influence the reliability estimates.
